# Deep learning framework for prediction of infection severity of COVID-19

**DOI:** 10.3389/fmed.2022.940960

**Published:** 2022-08-17

**Authors:** Mehdi Yousefzadeh, Masoud Hasanpour, Mozhdeh Zolghadri, Fatemeh Salimi, Ava Yektaeian Vaziri, Abolfazl Mahmoudi Aqeel Abadi, Ramezan Jafari, Parsa Esfahanian, Mohammad-Reza Nazem-Zadeh

**Affiliations:** ^1^Department of Physics, Shahid Beheshti University, Tehran, Iran; ^2^School of Computer Science, Institute for Research in Fundamental Sciences (IPM), Tehran, Iran; ^3^Research Center for Molecular and Cellular Imaging, Tehran University of Medical Sciences, Tehran, Iran; ^4^Department of Medical Physics, School of Medicine, Mashhad University of Medical Sciences, Mashhad, Iran; ^5^Department of Medical Physics and Biomedical Engineering, Tehran University of Medical Sciences (TUMS), Tehran, Iran; ^6^Department of Radiology, Health Research Center, Baqiyatallah University of Medical Sciences, Tehran, Iran

**Keywords:** COVID-19, deep learning, infection segmentation, lobe segmentation, CT scan, severity score

## Abstract

With the onset of the COVID-19 pandemic, quantifying the condition of positively diagnosed patients is of paramount importance. Chest CT scans can be used to measure the severity of a lung infection and the isolate involvement sites in order to increase awareness of a patient's disease progression. In this work, we developed a deep learning framework for lung infection severity prediction. To this end, we collected a dataset of 232 chest CT scans and involved two public datasets with an additional 59 scans for our model's training and used two external test sets with 21 scans for evaluation. On an input chest Computer Tomography (CT) scan, our framework, in parallel, performs a lung lobe segmentation utilizing a pre-trained model and infection segmentation using three distinct trained *SE-ResNet18* based *U-Net* models, one for each of the axial, coronal, and sagittal views. By having the lobe and infection segmentation masks, we calculate the infection severity percentage in each lobe and classify that percentage into 6 categories of infection severity score using a k-nearest neighbors (k-NN) model. The lobe segmentation model achieved a *Dice* Similarity Score (DSC) in the range of [0.918, 0.981] for different lung lobes and our infection segmentation models gained DSC scores of 0.7254 and 0.7105 on our two test sets, respectfully. Similarly, two resident radiologists were assigned the same infection segmentation tasks, for which they obtained a DSC score of 0.7281 and 0.6693 on the two test sets. At last, performance on infection severity score over the entire test datasets was calculated, for which the framework's resulted in a Mean Absolute Error (MAE) of 0.505 ± 0.029, while the resident radiologists' was 0.571 ± 0.039.

## 1. Introduction

Coronavirus 2019, or COVID-19, is a pandemic infectious disease that was reported in December 2019 from Wuhan, China, following an outbreak of the acute respiratory syndrome virus SARS-CoV-2 (1–4). Common symptoms include fever, cough, shortness of breath, and lethargy. Muscle pain, sputum production, sore throat, nausea, and red eyes are some of the less common symptoms (5, 6).

Reverse Transcription Polymerase Chain Reaction (RT-PCR) test is considered the *de facto* standard for diagnosing COVID-19 (7). However, lack of resources and strict environmental requirements limit the rapid and effective testing and screening of suspects. Moreover, RT-PCR has been reported having a high false-negative rate and a low sensitivity (8, 9).

Artificial Intelligence (AI) has risen its position in helping solve healthcare challenges in recent years. By involving a large amount of data, using advanced deep learning algorithms, and utilizing modern GPUs, exemplary achievements have been made in the fields of image classification and image segmentation (10–12). AI algorithms have demonstrated equal if not higher performance in medical image diagnosis in recent years while being fast and utilizable in pandemic situations. For example, Qin et al. (13), Ardila et al. (14), Nash et al. (15), Liao et al. (16), Mei et al. (17), Zhu et al. (18), and Xie et al. (19) have introduced algorithms with very successful and reproducible performance levels on pulmonary diseases such as tuberculosis and lung nodules and for lung cancer screening. With enough hardware resources, these algorithms could perform on a very large scale.

Machine learning and deep learning models have directly challenged the Coronavirus and have been successful in diagnosing the virus with high accuracy and reducing manpower efforts (20–22). Several deep learning models have been developed to diagnose COVID-19 from chest X-Ray or CT scans (17, 23–25). Furthermore, the fields of machine learning and data science have also been used effectively to diagnose and prognoses the virus and predict its outbreaks (26–28). Finally, it has been shown that a model serving as an assistant to the radiologists is successful in diagnosing the virus and in addition to increasing the expert's sensitivity, is also effective in increasing their specificity (9).

One of the applications of deep learning in computer vision, and in particular medical imaging, is segmentation, in which trained models automatically separate parts of the image (29–31). Several pieces of researches have been conducted in the field of infection segmentation and measuring the volume of infection for COVID-19 (32–35).

Pulmonary lobe segmentation has proven to be an important task as knowing the location and distribution of pulmonary diseases such as emphysema and nodule can be integral in determining the most suitable treatment. To this end, models for lobe segmentation have also been developed (36–39). As an example, Hofmanninger et al. (40) has introduced a well-performing lobe segmentation model trained on patients diagnosed with COVID-19.

Measuring the severity of a lung infection in patients with COVID-19 is a very challenging task and is an important prognosis for a patient's treatment process. Several diagnosis methods, some specifically designed to assess the severity of the disease, have been proposed based on the observation that the imaging biomarkers in patients with COVID-19 such as Ground-Glass Opacity (GGO) and infection-associated thickening of the interlobular septa are similar (41–44). Additionally, several similar works have tried to predict the severity of lung infection with the help of neural networks and machine learning models (42, 45–47).

In this work, we propose a framework for accurate lung lobe infection severity prediction. We do so by collecting and labeling 253 chest CT-scan from three hospitals, involving 59 labeled external scans from public datasets, and with the help of several deep learning and machine learning models. We perform lobe and infection segmentation to predict lung lobes' infection severity percentage and classify that percentage into an infection severity score as the framework's final output.

In short, the main advantages and novelties of our work are as follows:

- In contrast to previous works, we isolate and predict the infection severity within each lung lobe.
- Our research involves three datasets collected internally, as well as two additional public datasets for more verity and range.
- The data involved was labeled for lobe segmentation, infection segmentation, and infection severity. In addition, our test sets were labeled with more precision and accuracy.
- Separate distinct deep learning models were trained for the lobe and infection segmentation tasks and a machine learning model was trained for predicting the infection severity score.
- For a more thorough evaluation, our train and test data are from different hospitals.
- Our framework's performance was comprehensively compared with the performance of resident radiologists, for which our framework outperformed.
- In a post-COVID world, this framework is usable and extendable to other pneumonia infections.

The rest of this paper is structured as follows; In the second section, we explain the data involved and the used methods for this research in detail. In the third section, we outline our model's results for lobe segmentation and infection segmentation and present the framework's performance in infection severity prediction, in addition to comparing our performance result's with that of human experts. Finally, in the last section, we conclude with a discussion on the results, our work's limitations and challenges, and possible future directions.

## 2. Data and method

In this paper, we first adopt a deep learning model to detect the lobes. Next, by utilizing the subset of the data with an infection mask label to train an infection segmentation model, we finally combine the results with a k-Nearest Neighbors (k-NN) model to reach a final infection severity prediction.

In this section, we start by describing the different datasets involved and an overview of the distinct used pre-processes in Section 2.1. In Sections 2.3, 2.4, we discuss the adopted deep learning models and infection severity prediction methods in each lobe, respectively. Section 2.5 discloses our evaluation methods and criteria. Finally, we conclude the section with an explanation for our methods of evaluating radiologists and residents in Section 2.6.

### 2.1. Datasets

Three datasets for training and two datasets for evaluation were involved in this work. A 232-case cohort was collected from the Ghiassi Hospital database and the *MosMedData* (48, 49) datasets that are available publicly. Two smaller sets of *External Test 1* and *External Test 2* from Kasra Hospital and Imam Hossein Hospital, respectively, were involved in the model's final evaluation. All three aforementioned centers are located in Tehran, Iran. A complete taxonomy of the data can be found in Table 1.

**Table 1:** Involved datasets with their respective label count.

	**Dataset**	**Scans**	**Scans with** **Inf-Seg Label**	**Scans with** **Lobe-Seg Label**	**Scans with** ** Lobe-Inf severity label**	**Slice thickness**
Train and validation	Our dataset	232 (30157)	60 (8428)	54 (7014)	232 (30157)	*2 mm*
	MosMedData	50 (2049)	50 (2049)	-	-	*8 mm*
	Medical_Seg	9 (829)	9 (829)	-	-	*1-6 mm*
Test	*External Test 1*	11 (481)	11 (481)	11 (481)	11 (481)	*8 mm*
	*External Test 2*	10 (1443)	10 (1443)	10 (1443)	10 (1443)	*2 mm*

Each set possibly includes a limited count of infection segmentation, lobe segmentation, or lobe infection severity labels.

Three distinct scan labels are included in this work. The first is for infection segmentation and was extracted from 81 subjects. The second is for lobe segmentation which is extracted from a chest CT scan (masked with 5 colors; outside of the lung masked with black) in order to isolate each lobe; and the third is for lobe infection severity which we explain next.

According to the systematic report standard found on the *Radiologyassistant* (50) website, each severity percentage is categorized from 0 to 5. This can be found in Table 2. Radiology experts, relying on their experience and expertise in interpreting chest CT scans and their knowledge of the cross-sectional anatomy of different areas of the lung, have performed the lung lobe divisions and visually estimated the infection-resulted severity, which were present in a myriad of forms notably GGO and consolidation, in each lobe lattice with percentage and standard systematic report.

**Table 2:** Lobe infection severity percentage categorized by the number of points.

**# Points Present in the Infection**	**0**	**1**	**2**	**3**	**4**	**5**
Infection severity percentage	0%	<5%	5–25%	25–50%	50–75%	75–100%

In this research, 2 radiology residents generated systematic reports for all the scans in the study by referring to Table 2. Moreover, the *External Test* sets *1 and 2* scans that were labeled by two radiology residents were rectified by two senior radiologists each with more than 10 years of experience.

Lobe segmentation was performed semi-supervised by inputting the DICOM of scans into the *3D Slicer* software. Within the *Interactive Lobe Segmentation* section, the *Chest Imaging Platform (CIP)* module is used to generate the *Label Map Volume*, in complement with a *Fisser Volume* file, for the selected scans. A Gaussian filter is applied to enhance the lobe segmentation performance. Other parameters, such as dimensions are chosen by an expert.

By marking several points on the generated *Fisser*s in different scan views, especially sagittal, all five lobes are segmented and distinctly colored. The resulting export is then evaluated for authenticity by two radiology experts and if needed, is rectified by a skilled technician (rectification follows the standard procedure).

The Ghiassi Hospital cohort scans were taken using a TOSHIBA 16 CT scan machine. Each scan was taken with a low-dose setting and has a slice thickness of 2 *mm*. The 232-case cohort overall includes 30,157 axial view slices, containing 60 scans with infection segmentation and 54 with lobe segmentation labels. The entire cohort is labeled with lobe infection severity labels. The age and sex distribution of cohort subjects can be found in Supplementary Figure S1.

As lobe segmentation and infection labeling is a time-consuming process, it was performed restrictively and to necessity by two resident radiologists. The resulting set was involved in the training and validation of the machine learning model. Two additional sets, *External Test 1* and *External Test 2* were collected for final evaluation. The *External Test 1* includes 11 scans from patients from Kasra Hospital, Tehran, Iran, captured on a General Electric (GE) CT scan machine with 120 *KV* and 130 *MA* parameters and 7 *mm* slice thickness, totaling 481 *2D* slices. The *External Test 2* contains 10 scans from patients from Imam Hossein Hospital, Tehran, Iran, captured with a low-dose setting and 2 *mm* slice thickness. The obtained scans were in an axial view, each unprocessed slice in all the datasets was 512 × 512, and each pixel had a dimension of 0.76 × 0.76*mm*. Labeling for the two test sets was performed manually by two resident radiologists and subsequently revised by two expert senior radiologists.

Detailed information on the two public datasets involved in this research, *MosMedData* and *Medical_Seg*, are brought in Morozov et al. (48) and Jenssen (49). From the overall combination of these two sets, 80% was used for training and 20% for the validation of the framework.

Charts on the number of normal and infected slices, distribution of infection severity in scan slices, and sample count in each 6 class for different degrees of infection separated by lobe and for the training, validation, and test sets are described in Supplementary Figures S1–S7.

All collected data from hospitals in this research has been anonymized, official permissions have been obtained from the relevant department heads or supervisors, written consents were taken from all the participating patients, and the ethical license of IR.SBMU.NRITLD.REC.1399.024 was obtained from the Iranian National Committee for Ethics in Biomedical Research.

### 2.2. Data pre-processing

In this section, we describe the used pre-processes in detail; starting with the image pre-processing for infection segmentation models and following with the mask pre-processing for infection segmentation model outputs.

#### 2.2.1. Image pre-processing for infection segmentation models

In this pre-processing, first, a *3D* resizing on the coronal, axial, and sagittal views is applied using the zoom function (51) with regards to the *2D* cross-sections. For the *2D* slices, the *3D* image is initially resized to the following dimension:


$$\begin{array}{l} 512\times 512\times \text{number of slices}, \end{array}$$

but for extracting *2D* slices from coronal and sagittal views, the *3D* image, regardless of its initial dimensions, is resized to 256 × 256 × 256, and 256 slices are selected subsequently.

The window-level and window-width parameters were set to –600 and 1,500, respectively. This resulted in the image pixel intensity distribution to position between –1,024 (the lowest pixel value in all images) and 150. Next, this pixel range value was moved to the range [0, 255] using a linear transformation. The final result yielded three exact images from each *2D* slice. Lastly, as our models utilized weights obtained from training on the *ImageNet* [a dataset of 1.2 million images categorized to 1,000 classes (10)], all images were also additionally normalized to the same luminance of *mean* = [0.485, 0.456, 0.406] and *SD* = [0.229, 0.224, 0.225]. Data augmentation operations such as random white-noise addition and vertical-flip with probabilities 0.3 and 0.5, respectively, were also performed for the model's training.

#### 2.2.2. Infection mask pre-processing for training infection segmentation models

**Figure 1:**
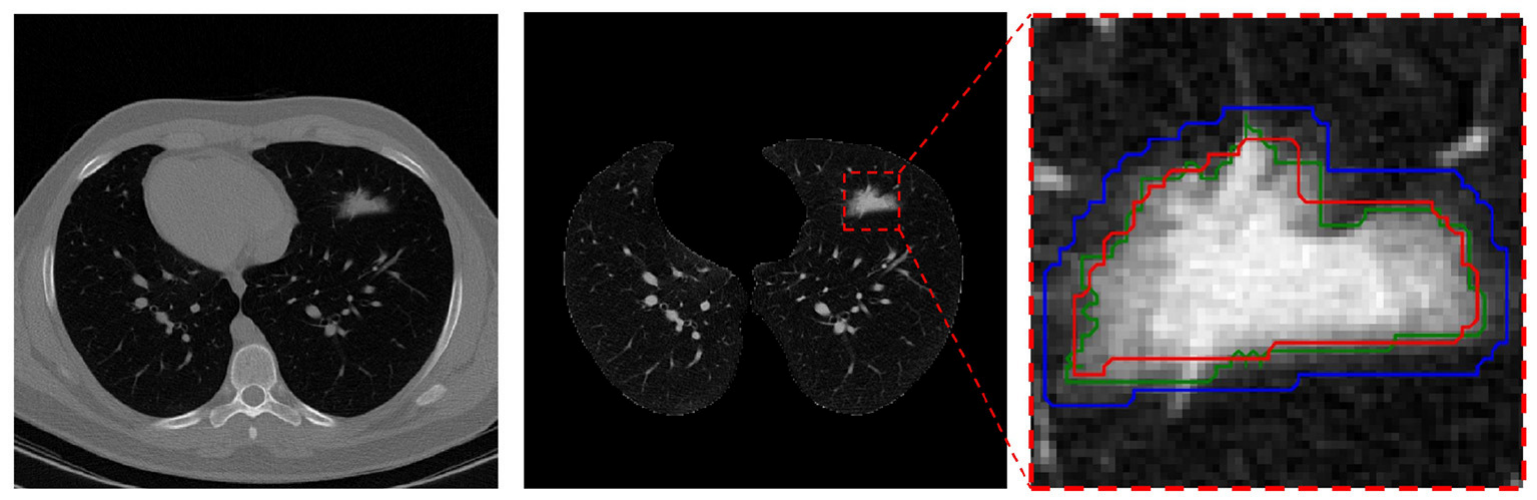
Mask pre-processing in the infection segmentation dataset. Left is the raw CT scan slice, middle is the lung image in the same slice (with infection in the upper-left lobe), and right is the segmented infection area in the image. In the right, the red contour depicts the radiologist manual mask edge, the blue contour is the dilated red contour, and the green contour s the rectified mask edge.

This pre-processing was only applied to the manual segmentation masks on the training and validation sets just before the model learning and its goal was to rectify manual mask edges. To this end, the manually masked area is enlarged using the Dilation (52) method to the point that it covers all of the infection areas initially left out. As seen in Figure 1, the red contour depicts the manual mask edge, while the blue contour shows the enlarged manual mask.

For the final mask, pixels contained within the blue contour that have a value within the 95% distribution range of all the pixels within the red contour are kept (and regarded as “infection” pixels) while the other non-relevant pixels within the blue contour are removed. The resulting final mask edge colored in green is also depicted.

This process helps with mask-edge correction by removing “normal” (for air, *etc*.) and very bright (organ or bone) pixels from the initial area and adding any left-out small areas.

Finally, this pre-processing method was carefully and manually reviewed on a large sample set ad after evaluation from an expert radiologist, its resulting rectified masks replaced undesirable ones. The test set rectified masks went through a more thorough and manual evaluation process under two senior radiologists.

### 2.3. Deep learning models

The deep learning models adopted by this research perform two tasks; lobe segmentation and infection segmentation. In this section, we explain each in detail.

#### 2.3.1. Lobe segmentation deep learning models

For this task, the deep learning models described in Hofmanninger et al. (40) were adopted. These models include *U-Net* (53), *ResU-Net* (54, 55), *Dilated Residual Network-D-22* (56), and *Deeplab v3+*(57). From these models, the *U-Net R231* (40) model performed the best on our test set data, for which the results can be found in Section 3, and was thereby selected for our framework. The output of the selected model would segment a lung image to its five lobes using 2D slices in the axial view and as its results were sufficiently desirable, we refrained from developing our custom model for the lobe segmentation task. The model performance on our dataset is discussed in Section 3.

#### 2.3.2. Infection segmentation deep learning models

For this task, we adopted several *FPN* (58), *PSPNet* (59), *LinkNet* (60), *U-Net* (53), and *U-Net++* (61) models based on their respective *EfficientNet* (62), *SE-ResNet* (63), *etc*. architectures. As the performance of these models was closely similar, a detailed discussion on the subject was omitted.

In the end, an *SE-ResNet-18* based *U-Net* model was selected for our framework. Three separate instances of the model are used. The first is for infection segmentation on *2D* slices of the axial view. The input for this model instance, with dimensions 512 × 512 × 3 (3 is for the RGB channels) is the largest it can be in order to extract as many features as possible. The second and third model instances are used similarly for the coronal and sagittal views, respectively, but with a 256 × 256 × 3 input dimension. Overall, as previously discussed in Table 1, 119 CT scans with an infection segmentation label were used for the training and validation of these models.

The encoder part of these model was initialized with pre-trained *ImageNet* weights and their last layer activation function is *Sigmoid*. Additionally, the prediction layer of the models includes an infection detection channel. The cost function used for all the models is:


$$\begin{array}{l} Loss=(1-\frac{2\times P{\cap^{\text{​}}}^{\text{​}}T}{P+T})\\ \;+(-\frac{1}{N}\overset{N}{\underset{i=1}{{\sum^{\text{​}}}^{\text{​}}}}[T_{i}\log(P_{i})+(1-T_{i})\log(1-P_{i})]), \end{array}$$

where *T* and *P* are the pixel label and prediction, respectively, the first RHS term is the *Dice* loss, and the second RHS term is the binary cross-entropy error function.

The *Keras* package was used for everything deep-learning related and the *NiBabel* (51) and *PyDicom* (64) packages were used for working with medical images. All of the development and evaluation processes were executed with the Python programming language version 3.7.9.

### 2.4. Framework overview

**Figure 2:**
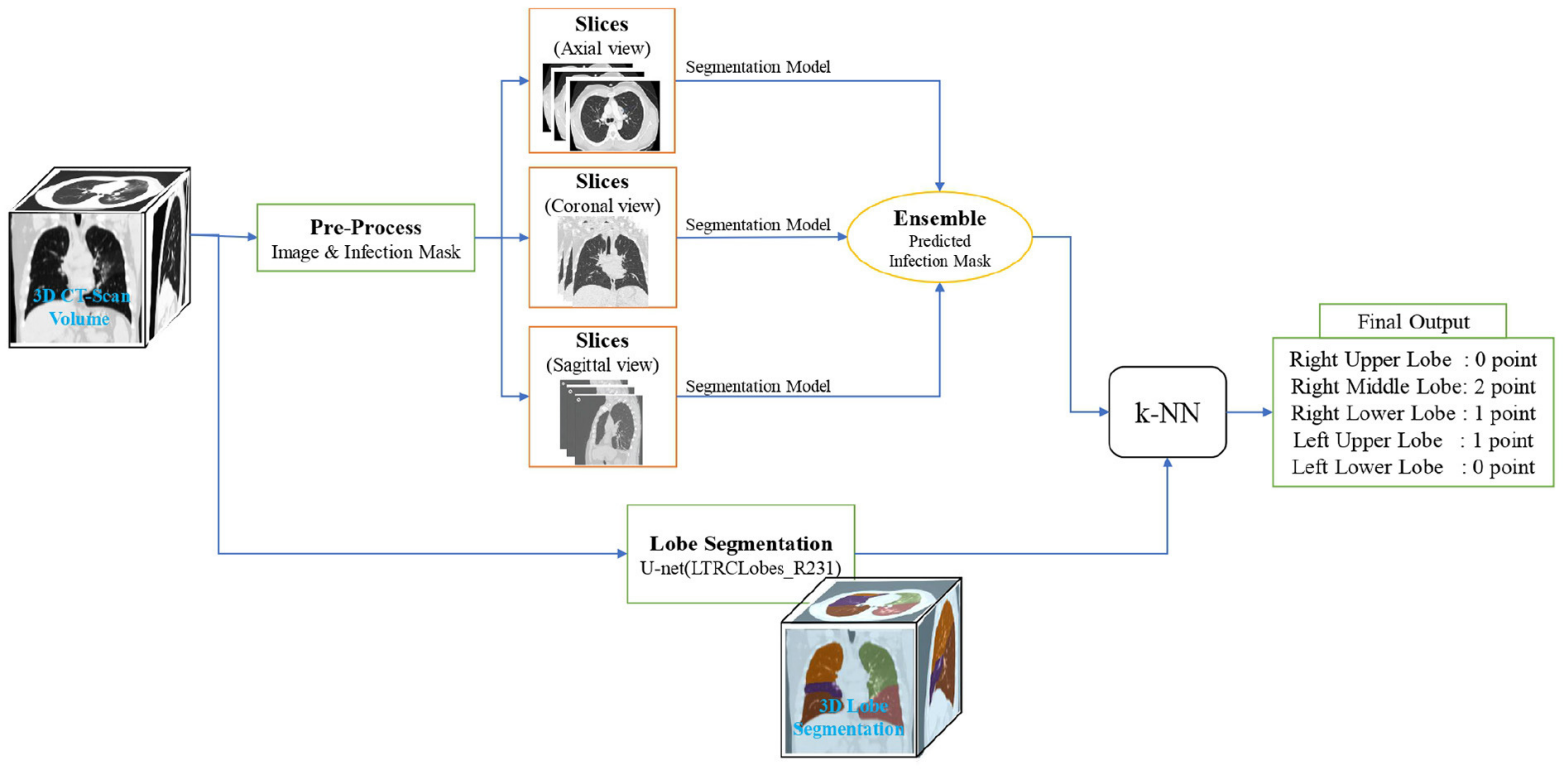
Overview of the lung lobes infection severity prediction framework. An input image is simultaneously given the lobe segmentation and infection segmentation models. Then, by combining the output of these two models, the infection percentage of each lobe is predicted and given as the input of the k-NN model to predict the severity of infection in terms of the 6 classes of infection severity for all the 5 lobes.

In this section, we break down our framework into its principal machine learning and deep learning components, as showcased in Figure 2, and characterize the final output construction methodology.

On an input, we first utilize the lobe segmentation model to determine what lobe each pixel belongs to (or if it does not belong to any). Next, we employ the three distinct *U-Net* models mentioned in Section 2.3.2 to obtain infection segmentation across the three different views. The outputs are then combined with a weighted-averaging ensemble learning technique (a weight of 5 for the sagittal and coronal views and a weight of 1 for the axial view) to produce a final infection segmentation for the framework input.

As the axial view in our data has a higher image resolution and will probably result in higher model accuracy, the used ensemble learning technique gives it a larger weight compared to the similar coronal and sagittal view weights. This assumption is studied in Section 3.

Next, using the ensemble model output, each lobe pixel is evaluated in terms of containing infection to determine the overall percentage of each lobe's pixels involved with infection. Finally, the lobes infection severity percentage is fed to a k-NN model to learn according to the label given by the specialist in order to classify the percentage into the 6 classes of infection severity predicted by the experts.

The k-NN model is used since the expert did not necessarily calculate the infection severity by counting the pixels, but based on the experience in the field. The overall structure of the flow described in this section is showcased in Section 3.

### 2.5. Statistical inference

In order to more reliably evaluate our results, we chose different criteria for different parts of our research. Our results are also reinforced by performing the same evaluations on a team of experts.

By incorporating error propagation and Bayesian statistics, the marginalized confidence region is calculated at a 95% level for each output. The prediction result significance is determined by calculating the *p*-value statistics systematically. In order to achieve a conservative decision, the 3σ significance level is considered.

The lobe and infection segmentation models were evaluated with the following criteria on the scan:


$$Dicescore=\frac{2\times P\cap^{\text{​}}T+\epsilon }{P+T+\epsilon }$$

where ϵ is a small value, added to prevent the denominator from becoming zero.

At last, the Mean Absolute Error (MAE) was used to evaluate the infection severity prediction performance.

### 2.6. Experts evaluation

As the train and validation sets of our data were manually labeled by two resident radiologists and were therefore prone to error, and since the segmentation labeling and infection severity categorization tasks were time-consuming and the data for it was not present in radiology reports by default, we opted to involve the data with the least possible amount of error in our framework evaluation. To this end, the *External Test* sets *1 and 2* were collected which were also used to evaluate our experts.

For the evaluation of experts, which included two resident radiologists, we asked them to label each case with segmentation and infection severity labels. Labels from one expert were regarded as the ground truth while labels from the other as a prediction. The metric similarity between the two experts is reported as the minimum expert accuracy. It is important to note that the labels produced by the two resident radiologists were rectified by the two senior radiologists for the final test set. This difference between the initial labels and the rectified labels is why the expert evaluation metric is accompanied by a bias value.

## 3. Results

In this research, overall 291 CT scans were involved in training and validation and 21 CT scans for final evaluation. This section starts off and continues with the evaluation results for the lobe segmentation and infection segmentation models, respectively, and closes with the framework performance evaluation and its comparison with that of experts.

### 3.1. Lobe segmentation results

**Figure 3:**
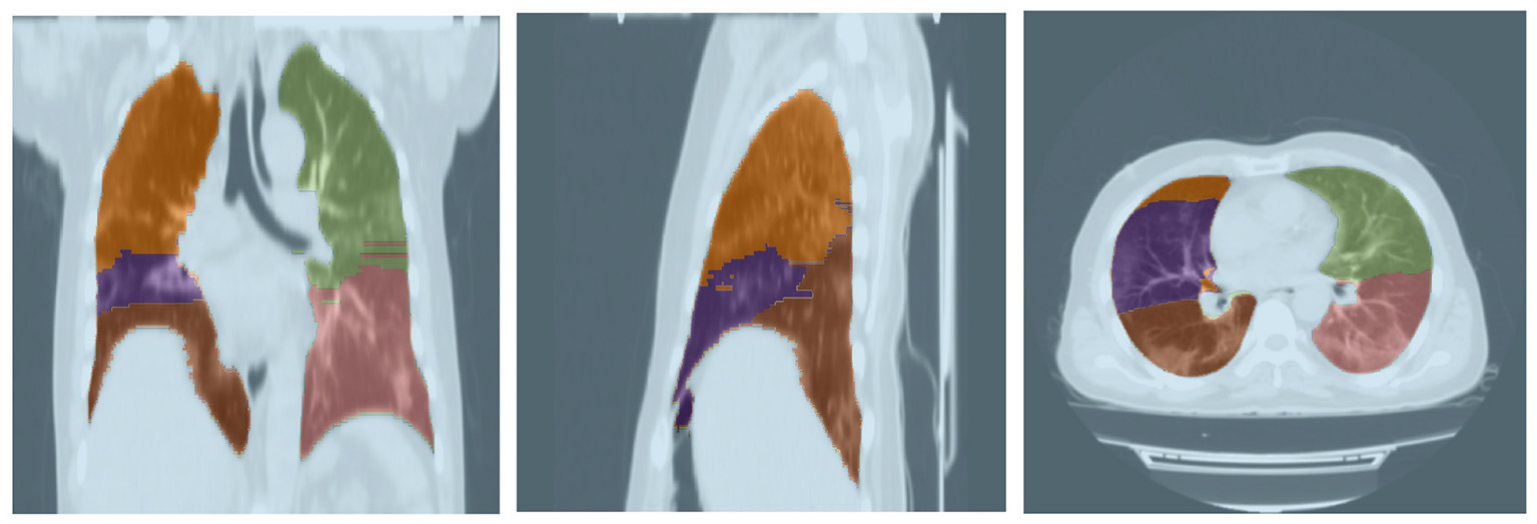
Lobe segmentation from three different views.

For this task, a pre-trained model from Hofmanninger et al. (40) was adopted as its performance was sufficiently desirable. This model was evaluated on 21 CT scans based on the *Dice* score for which the results are shown in Table 3. Furthermore, a sample output of this model for segmenting a lung's lobes is presented in Figure 3.

**Table 3:** Average *Dice* score of the lobe segmentation model on 21 CT scans.

	**Upper right**	**Middle right**	**Lower right**	**Upper left**	**Lower left**
Dice score	0.969 ± 0.075	0.918 ± 0.171	0.958 ± 0.101	0.980 ± 0.094	0.981 ± 0.086

### 3.2. Lobe infection severity prediction

Sixty scans from our own dataset and two 50 and 9 scan sets from the involved public datasets, all with infection segmentation labels, were used to train three *U-Net*based *SE-ResNet18* for the axial, coronal, and sagittal views and their outputs were combined using a weighted-average ensemble learning technique to gain a final infection segmentation result.

These models were evaluated on the validation and *External Test* sets *1 and 2* using *Dice* score and the final ensemble performance results can be seen in Table 4.

**Table 4:** *Dice* score of the infection segmentation models and their comparison with the performance of two resident radiologists.

**Set**	***Dice* (axial)**	***Dice* (coronal)**	***Dice* (sagittal)**	***Dice* (ensemble)**	**Res.1 vs. Res.2**
Validation	0.7312 ± 0.0423	0.7122 ± 0.0452	0.7191 ± 0.0509	0.7413 ± 0.0403	-
Test 1	0.7167 ± 0.0345	0.6386 ± 0.0387	0.6498 ± 0.0330	0.7254 ± 0.0341	0.7281 ± 0.0390
Test 2	0.7017 ± 0.0354	0.6582 ± 0.0411	0.6475 ± 0.0361	0.7105 ± 0.0399	0.6693 ± 0.0544

As the results demonstrate, the infection segmentation *Dice* score in the coronal and sagittal views are lower than the axial view, for which the ensemble performance of the three views barely matches. Moreover, the two resident radiologists' *Dice* score is on average lower than our framework. As the validation set was not labeled by both radiologists, the experts' performance over this set is not reported.

By incorporating the obtained lung lobe segmentation in the CT scan and having the infection segmentation model output, our framework predicts the overall infection severity with a number in the range [0, 100], which we report as a percentage.

**Figure 4:**
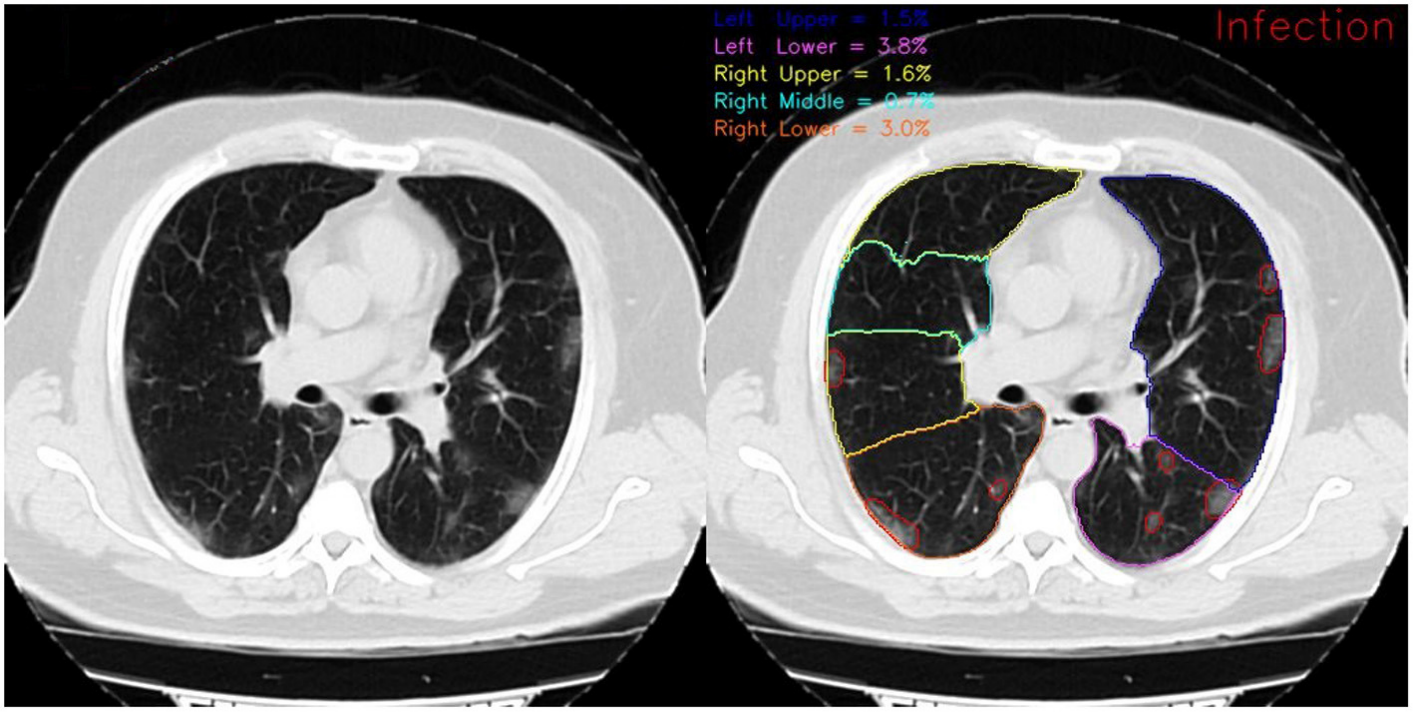
Framework output for a COVID-19 diagnosed patient scan on the 72^*nd*^ slice of the axial view. On the left, red contours show the infection and on the right, different colored contours show distinct lung lobes. The reported infection severity percentages correspond to each lobe and are reported the same for each slice.

In Figure 4, the framework's output for lobe segmentation, infection segmentation, and per-lobe infection severity prediction is showcased for a slice from a COVID-19 diagnosed patient axial chest CT scan.

But as the experts' prediction of the infection severity is purely visual and not by infected pixel count, our framework categorizes the infection severity percentage into 6 distinct levels utilizing a simple k-NN model with *k* = 7 with the combined outputs of the ensemble lobe and infection segmentation as its input to learn over infection severity score manually labeled by the experts. This k-NN model was eventually evaluated on 21 scans from the *External Test* sets *1 and 2*, for which the framework achieved an MAE error of 0.505 ± 0.029 on all lung lobes. A more detailed overview of the framework's performance can be seen in Table 5. For a more comprehensive evaluation, the MAE error was calculated for the two resident radiologists. The expert's error of 0.571 ± 0.039 was obtained, showing the better performance of our framework over expert human prediction.

**Table 5:** Model and expert (two resident radiologists) MAE error for different lung lobes over the 21 scans of our test sets.

**MAE**	**Upper right**	**Middle right**	**Lower right**	**Upper left**	**Lower left**	**All lobes**
Framework	0.429 ± 0.040	0.571 ± 0.051	0.571 ± 0.038	0.429 ± 0.031	0.524 ± 0.044	0.505 ± 0.029
Expert	0.619 ± 0.061	0.714 ± 0.054	0.667 ± 0.057	0.381 ± 0.032	0.476 ± 0.041	0.571 ± 0.039

As seen in Table 5, the prediction error for the right middle lobe is larger than other lobes due to this lobe being generally more difficult to predict for the framework (with the lowest *Dice* score of 0.918) and the experts.

**Figure 5:**
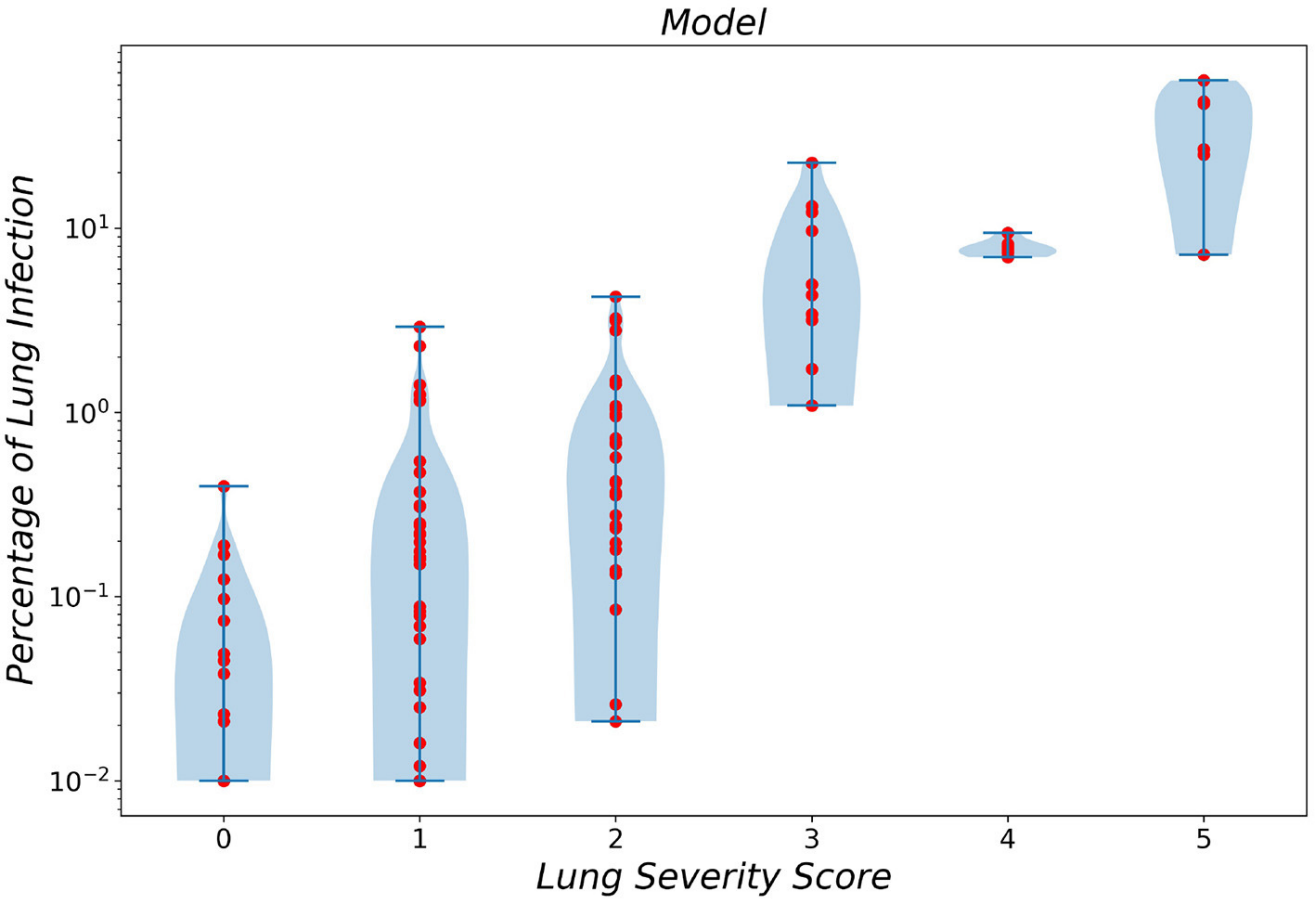
Violin plot of infection severity percentage predicted by the framework for 6 different classes.

To gain a better insight into the relationship between the predicted infection severity percentage by the framework and its corresponding infection severity score label, the prediction distribution over all 6 lobe infection severity classes are showcased as a violin plot in Figure 5. As depicted in the figure, the largest errors belong to classes 0 (normal) and 1 (infection severity lower than 5%). Notably, the infection severity percentages of these two classes are marginally close.

**Figure 6:**
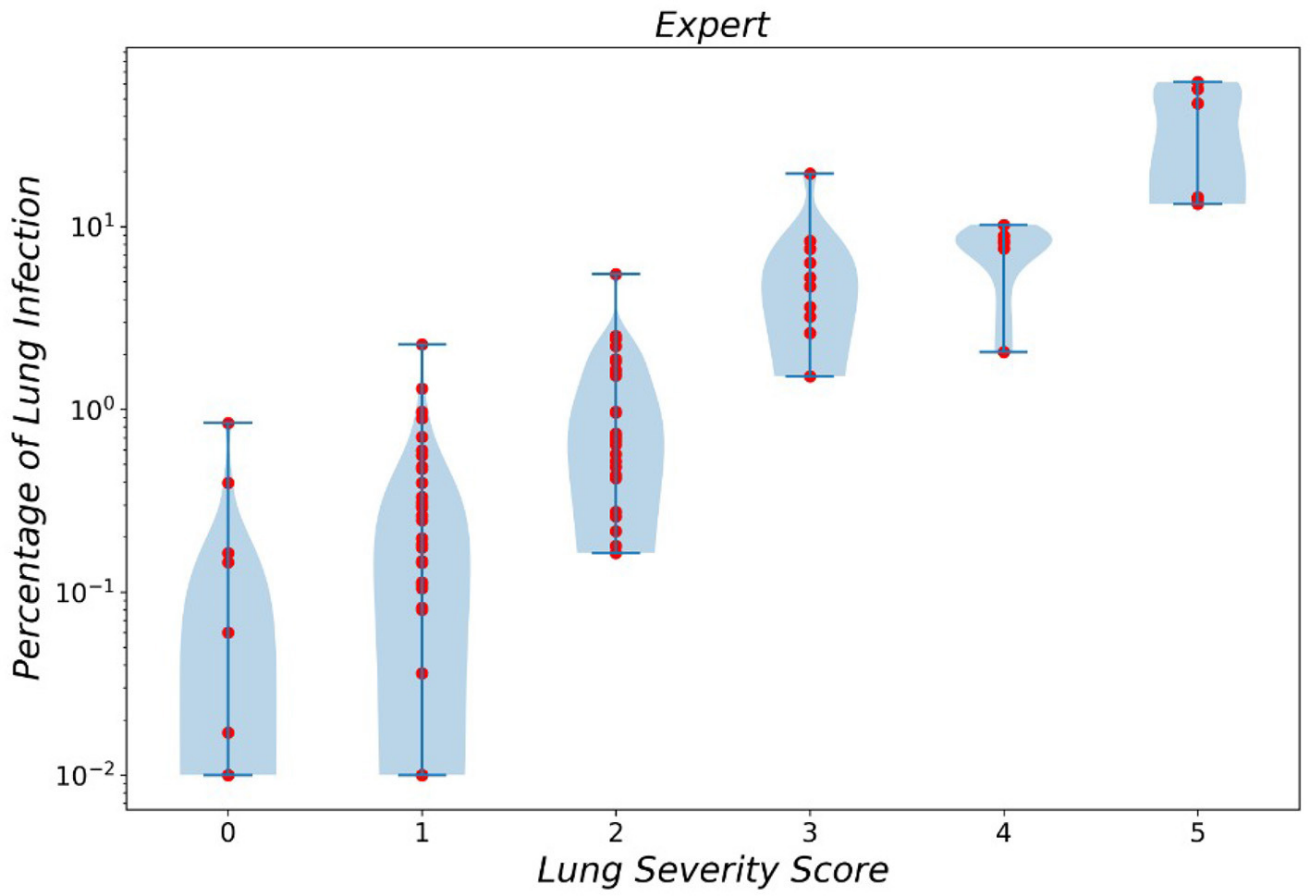
Violin plot of infection severity percentage predicted by experts for 6 different classes.

In a similar manner, the violin plot of labels produced by resident radiologists is shown in Figure 6, which clearly demonstrated the error produced by classes 0 and 1 while asserting the fact that diagnosing smaller infections is generally harder.

## 4. Conclusion and discussion

In this research, we collected chest CT scans of patients positively diagnosed with COVID-19 for training from one center and additional scans from two other hospitals with different imaging devices. Furthermore, two public datasets were also involved to include a wide range of data from different centers and several countries. These datasets were then labeled with their corresponding lobe segmentation, infection segmentation, and infection severity labels.

Next, we extracted lung lobes in each scan using our framework's lobe segmentation model. Thereafter, lung infection segmentation was performed utilizing several task-specific deep learning models in an ensemble manner. Finally, by measuring infection severity percentage and incorporating a k-NN model, each lung lobe was predicted for its infection severity score.

For lobe segmentation, we adopted the pre-trained *U-Net* model from Hofmanninger et al. (40). This model performed satisfactorily as it obtained a *Dice* score of 0.958 on all lung lobes except for the middle-right, for which the *Dice* score was 0.918. As diagnosing the middle-right lobe is generally more difficult for human experts, the model's lower *Dice* score in this lobe might have resulted from the labeling error.

For infection segmentation, the framework achieved a 0.725 *Dice* score, similar to that of the resident radiologists on the *External Test 1* set. The framework's performance on the *External Test 2* set was marginally better. Our model's performance and the fact that these two test sets were collected from two different centers with different imaging devices attest that our framework is robust to the imaging device configuration and parameters. Yet, as the results show, this claim did not hold for the human experts. Overall, our framework demonstrates performance at levels similar to that of at least a resident radiologist.

Finally, we compared the MAE error of infection severity prediction between our framework and a resident radiologist. On all the combined data from the two *External Test* sets *1 and 2* and over all the lung lobes, our framework achieves a lower error compared to the resident radiologist, which is shown in Table 5. Moreover, the correlation between infection severity percentage and infection severity score categorized in 6 levels was studied and showcased *via* violin plots for both our framework and the human experts. This study showed that the difference between classes 0 and 1 is marginally small and differentiating these two classes yields the largest error.

One of the previous works in the field, Ma et al. (32), developed an infection segmentation deep learning model with a *Dice* score on the right and left lungs equal to 97.7 and 97.3, respectively. Li et al. (33) managed to develop a deep learning model for the same task to gain a 0.74 *Dice* score, while the expert score in their study was close to their model at 0.76. Voulodimos et al. (35) developed an *FCN* and a *U-Net* model for infection segmentation with a *Dice* score peaking at around 0.65. Lastly, Abdel-Basset et al. (34) developed a novel model for learning on a small-sized labeled set denoted *Few-Shot Segmentation* with a *Dice* score of 0.80. An overview of the evaluation results in this section can be found in Table 6.

**Table 6:** Infection segmentation performance comparison over several sample sets against several similar works.

**Approach**	**Sample set count**	**Dice score**
Ours	140 CT scans	0.71 - 0.74
Li et al. (33 )	30 CT scans	0.74
Voulodimos et al. (35)	10 CT scans	0.65
Abdel-Basset et al. (34)	939 CT slices	0.80

The majority of similar research in the field aims to predict infection severity in the entire lung, while our work narrows down and isolates the prediction to each lobe. In a similar fashion (65), the author developed a Support Vector Machine (SVM) model utilizing the probability density function to classify lung lobe infection severity with an Area Under Curve (AUC) score in the range of [0.64, 0.87] on the validation set.

In our initial assessments for this research, we also experimented on 3D convolution models with a 3D-UNet model for the infection segmentation for which its lower performance results made us refrain from discussing it. However, the interested reader is encouraged to study and evaluate different models and methods for this task.

We used an Nvidia RTX 5000 graphics card for the computational portions of this work. For a CT scan of about 100–150 slices, our framework will perform lobe segmentation, infection segmentation, and infection severity prediction on all slices and finish up with an overall infection severity for each lung lobe in the scan in under 5 min. While doing the exact same task takes more than an hour for a human expert.

As for the limitations of this research, the biggest one involves data collecting. Labeling for lobe and infection segmentation is a rather time-consuming process. The fact that several licenses from multiple official bodies are required to collect the data in the first place adds to the time consumption of the data collecting process.

For this research, since the necessary grants were not provided at the time of data labeling, the labels were produced by the resident radiologists and only the *External Test* sets *1 and 2* were later labeled by senior radiologists. This resulted in us only being to evaluate the resident radiologists. Evaluating the senior radiologists with a thoroughness level matching the rest of the research required expert manpower beyond what we could manage to bring (at least 5 more senior radiologists).

Another limitation was the rather small size of level 4 and 5 infection severity samples in our datasets, which certainly hampered our framework's performance. In addition, a much larger dataset of normal scans is required to reduce the prediction error between classes 0 and 1.

A future improvement on this work might include involving more data from more centers with different devices and configurations in the models training. A study on the effect of different scan dosages is also beneficial. To address one of the other limitations of this work, data from patients with an age distribution that includes younger subjects will certainly improve the framework's comprehensiveness. Improving the error margin on the manual process of lobe and infection segmentation labeling and infection severity estimation would also help the framework's performance.

In addition, we observed instances of the model incorrectly recognizing pulmonary vessels around the umbilical cord area as infection (which would get worsen with noisy data). To address this, a more complex deep learning model trained on data that also has vessel segmentation is likely needed. The *pièce de résistance* would be a model that could predict the infection severity score of a chest CT scan, without requiring to perform lobe or infection segmentation.

To conclude, we developed a framework for infection severity prediction in lung lobes by involving several datasets, collected and public, and by utilizing multiple machine learning and deep learning models, in order to serve as a prognosis tool for the experts. Finally, we comprehensively evaluated our framework and compared its performance to experts to determine its benefit in helping the treatment process of patients.

## Data availability statement

The data analyzed in this study is subject to the following licenses/restrictions: The CT dataset has been collected in Ghiassi Hospital, Tehran, Iran. It cannot be shared publicly because of ethical restrictions and sensitive human study participant data. The anonymized (non-personally identifiable) data is, however, available from the Institutional Data Access through the Ethics Committee of Ghiassi Hospital for the researchers who meet the criteria to access the confidential data (contact the corresponding author at mnazemzadeh@tums.ac.ir). Requests to access these datasets should be directed to M-RN-Z mnazemzadeh@tums.ac.ir.

## Ethics statement

The studies involving human participants were reviewed and approved by Tehran University of Medical Sciences, IR.TUMS.IKHC.REC.1399.447. The patients/participants provided their written informed consent to participate in this study.

## Author contributions

MY: main contributor, design and implementation of machine learning algorithms, and writing the manuscript. MH: design and implementation of machine learning algorithms and revising the manuscript. MZ: data acquisition and manuscript preparation. FS, AY, and AM: data labeling and processing. RJ: radiologist, imaging reporting, and supervision of data labeling. PE: implementation of machine learning algorithms and revising the manuscript. M-RN-Z: definition of project, providing the fund, design of machine learning algorithms, and revising the manuscript. All authors contributed to the article and approved the submitted version.

## Funding

This project was partially funded by the Tehran University of Medical Sciences (TUMS), the International Campus (IC) Research Affairs with Research Code: 99-2-163-49030. The funding was merely for the data processing and modeling and did not include any open access publication fees.

## Conflict of interest

The authors declare that the research was conducted in the absence of any commercial or financial relationships that could be construed as a potential conflict of interest. The reviewer SK declared a shared affiliation with the author MY to the handling editor at the time of review.

## Publisher's note

All claims expressed in this article are solely those of the authors and do not necessarily represent those of their affiliated organizations, or those of the publisher, the editors and the reviewers. Any product that may be evaluated in this article, or claim that may be made by its manufacturer, is not guaranteed or endorsed by the publisher.

## Supplementary material

Supplementary Figure 1 Left: Age distribution of study subjects. Right: Sex distribution of study subjects.

Supplementary Figure 2 Number of normal and infected slices of the data used in the train and validation sets.

Supplementary Figure 3 Slice infection percentage in the train and validation sets.

Supplementary Figure 4 Sample count of different classes of infection severity for the 5 lung lobes and overall.

Supplementary Figure 5 Number of normal and infected slices of the data used in the test set.

Supplementary Figure 6 Slice infection percentage in the test sets.

Supplementary Figure 7 Sample count of different classes of infection severity in External Test sets 1 and 2.
