# Supplementary figures and images for: Deep learning framework for prediction of infection severity of COVID-19
Source: Front Med (Lausanne). 2022 Aug 17;9:940960. doi: 10.3389/fmed.2022.940960 (PMC9428758; doi:10.3389/fmed.2022.940960)

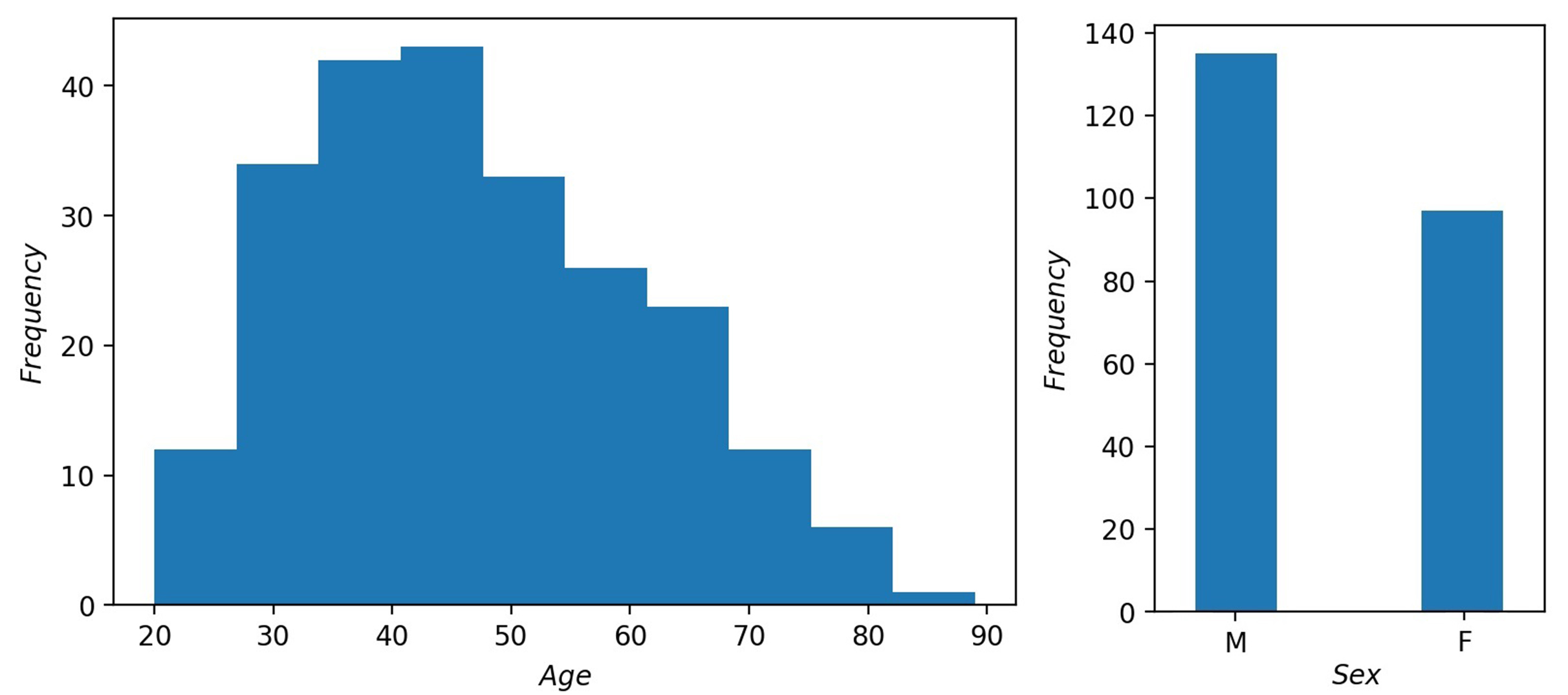

Supplement: Supplementary Figure 1 — Left: Age distribution of study subjects. Right: Sex distribution of study subjects. [file Image_1.jpg]

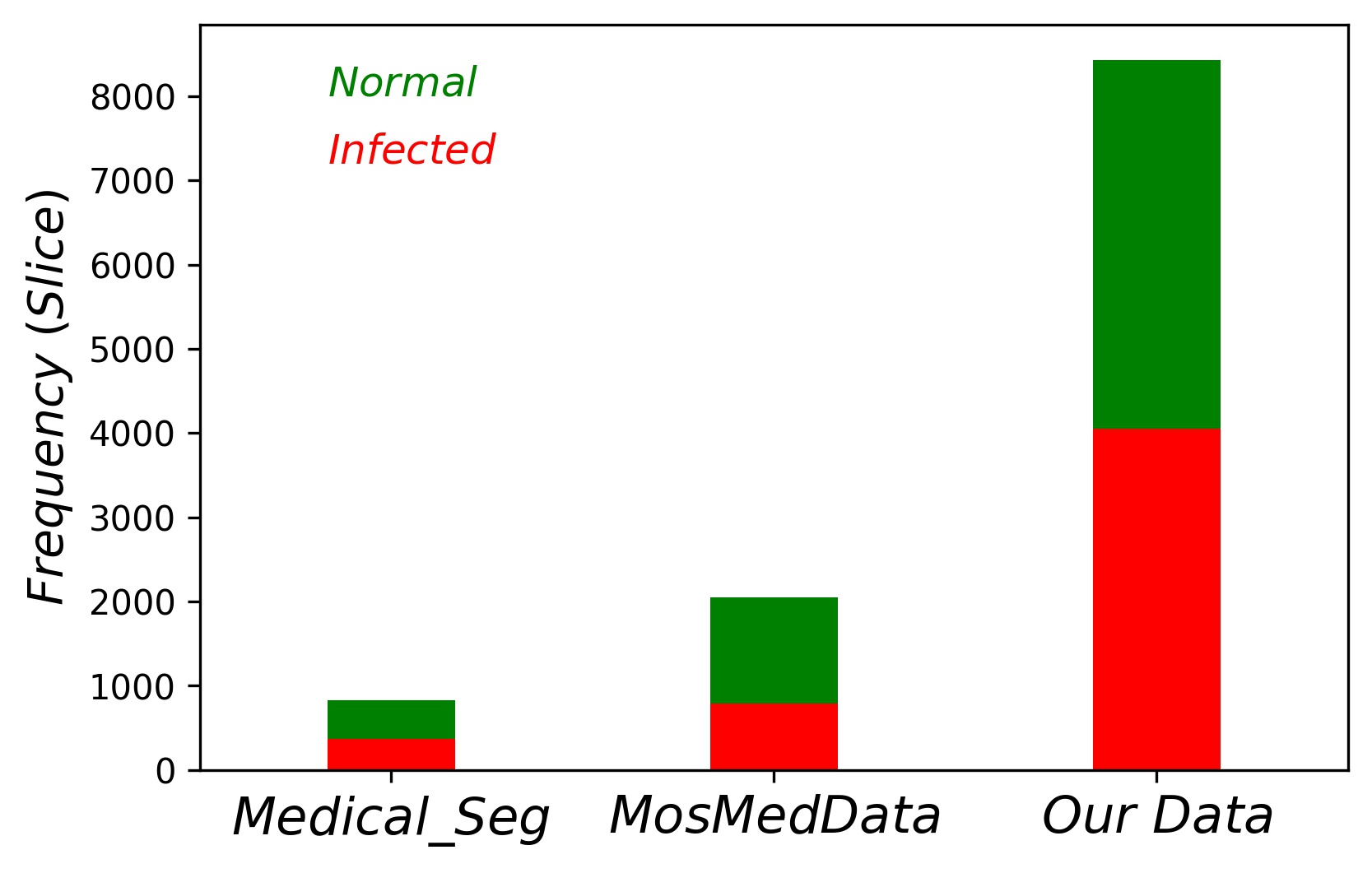

Supplement: Supplementary Figure 2 — Number of normal and infected slices of the data used in the train and validation sets. [file Image_2.jpg]

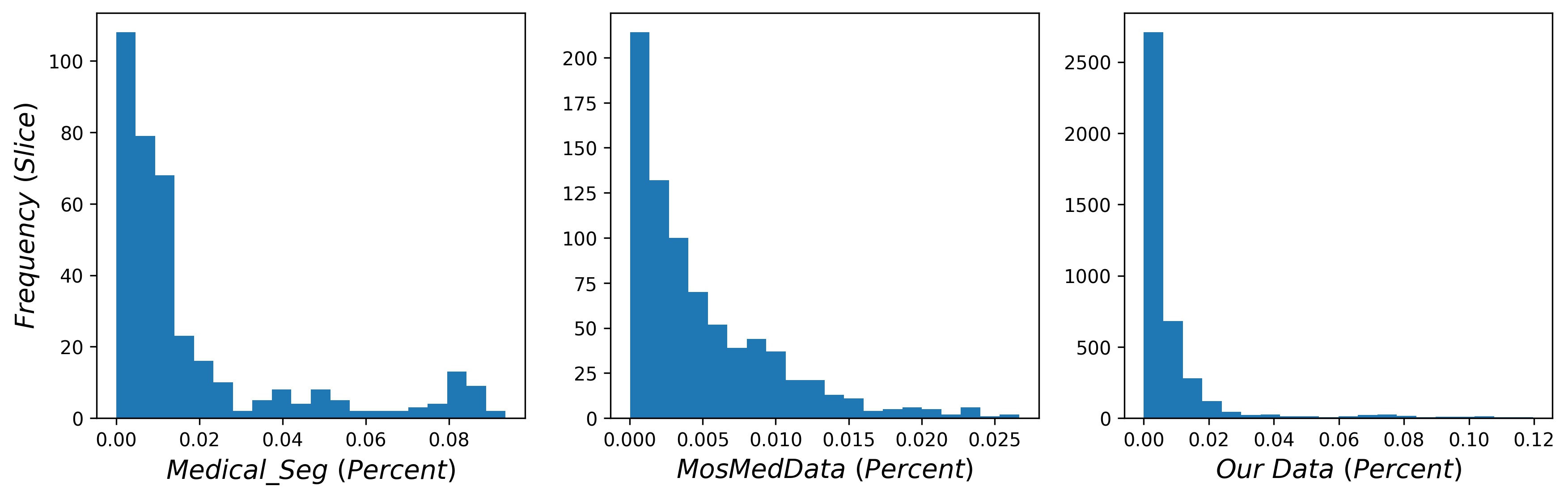

Supplement: Supplementary Figure 3 — Slice infection percentage in the train and validation sets. [file Image_3.jpg]

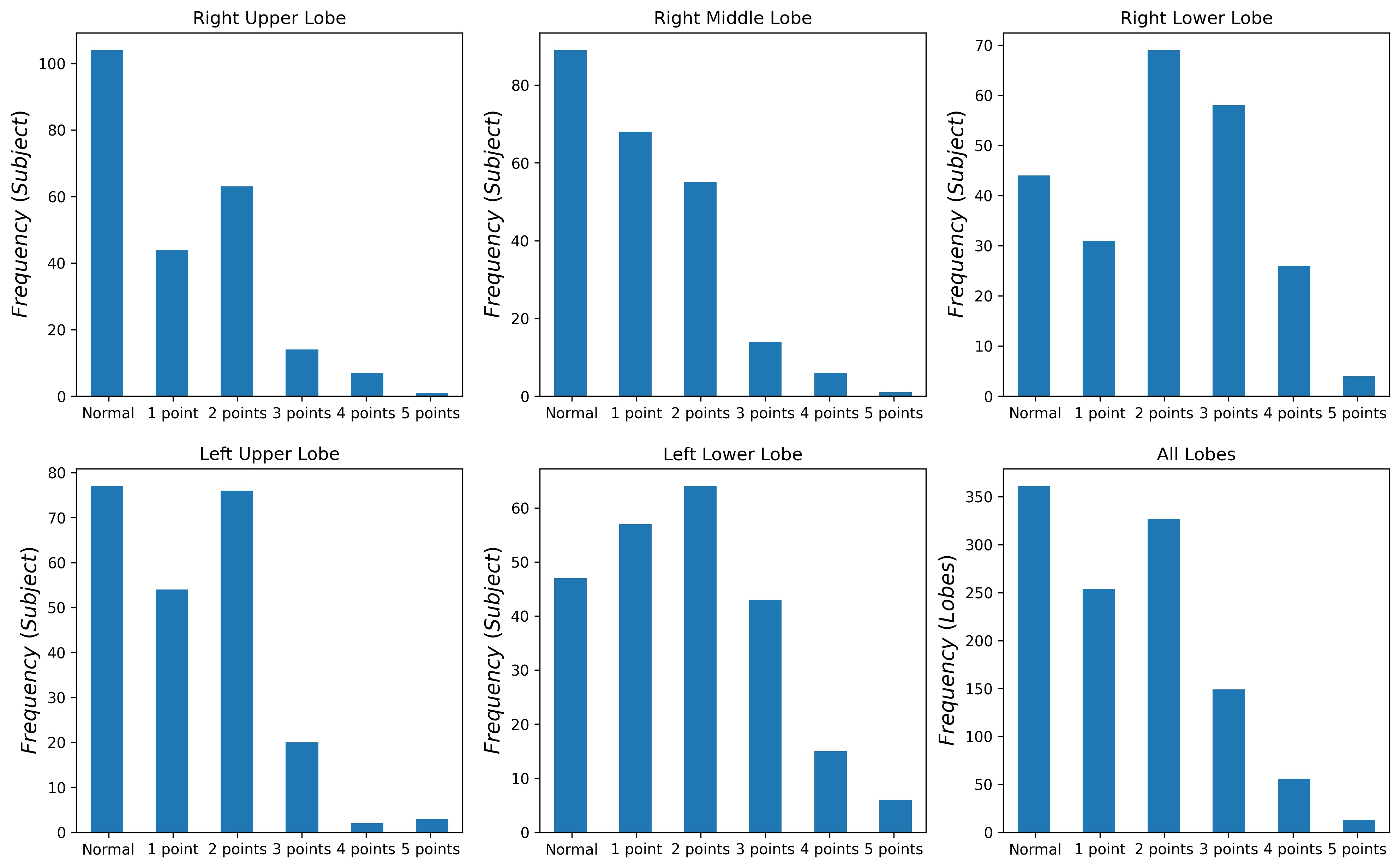

Supplement: Supplementary Figure 4 — Sample count of different classes of infection severity for the 5 lung lobes and overall. [file Image_4.jpg]

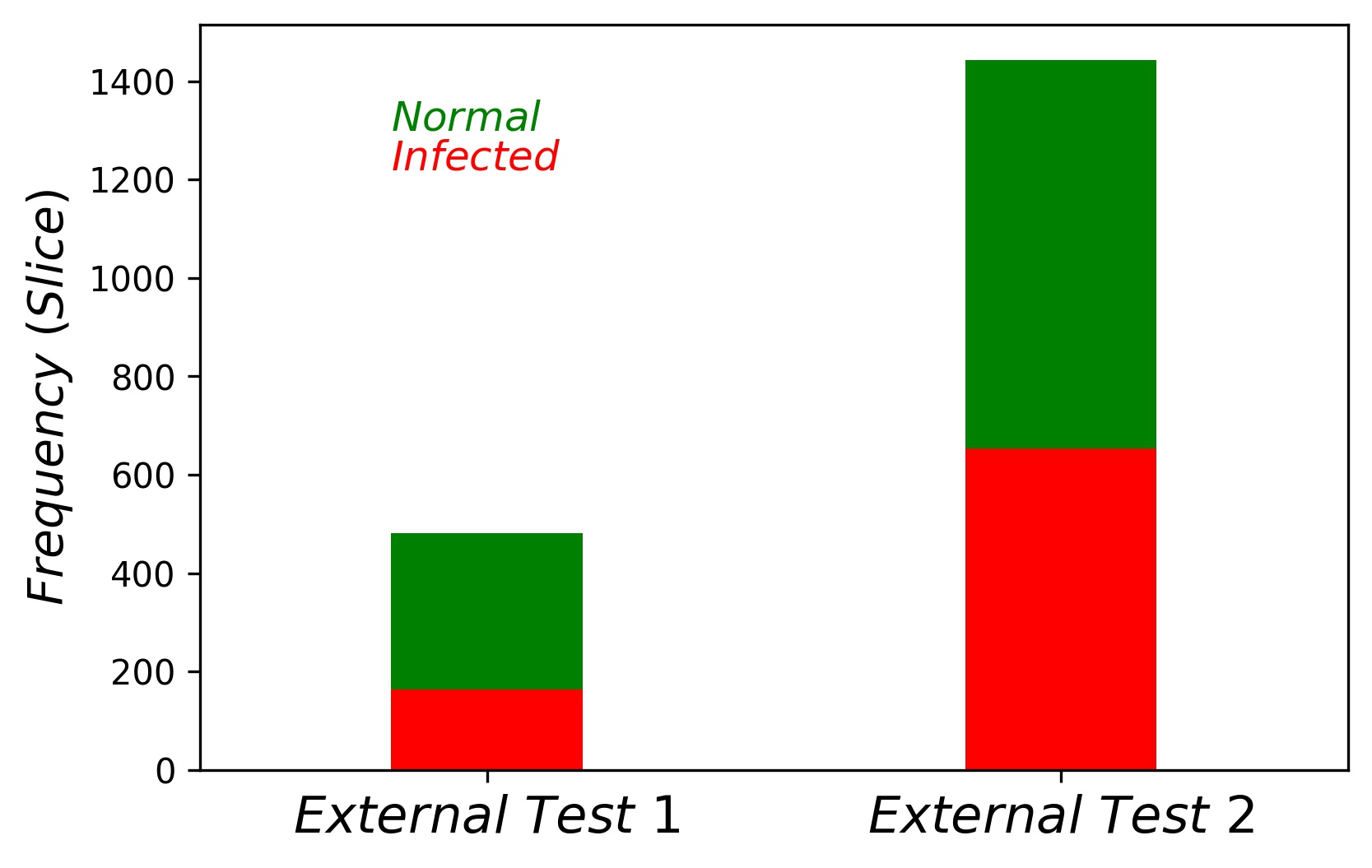

Supplement: Supplementary Figure 5 — Number of normal and infected slices of the data used in the test set. [file Image_5.jpg]

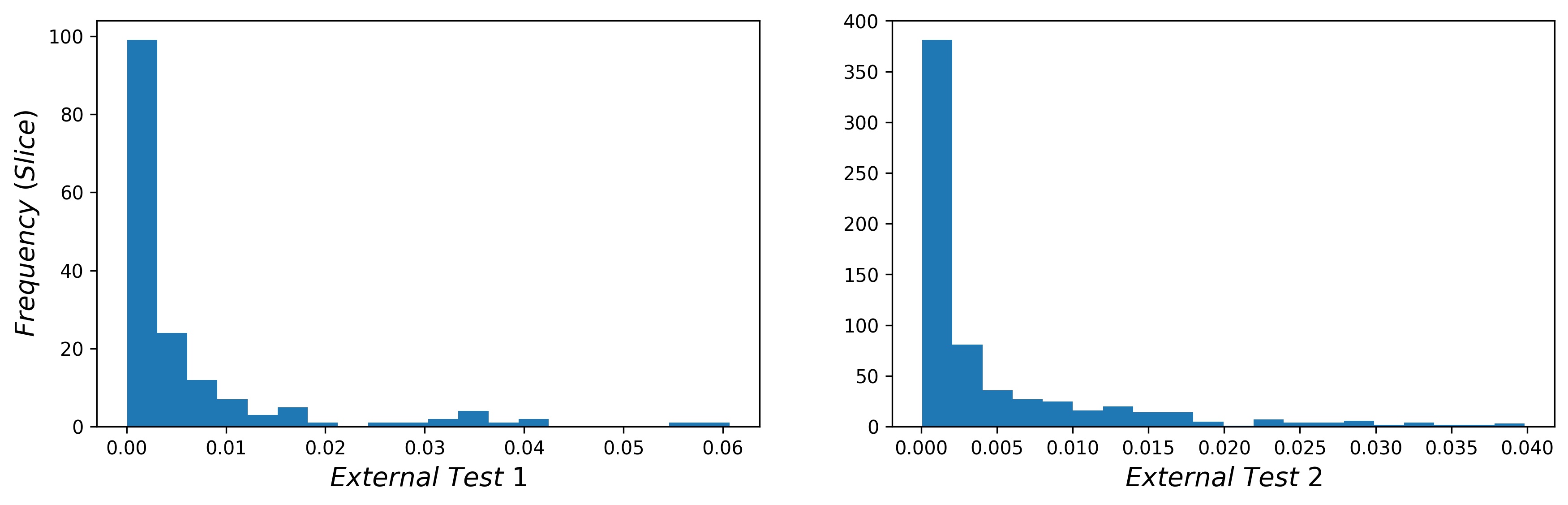

Supplement: Supplementary Figure 6 — Slice infection percentage in the test sets. [file Image_6.jpg]

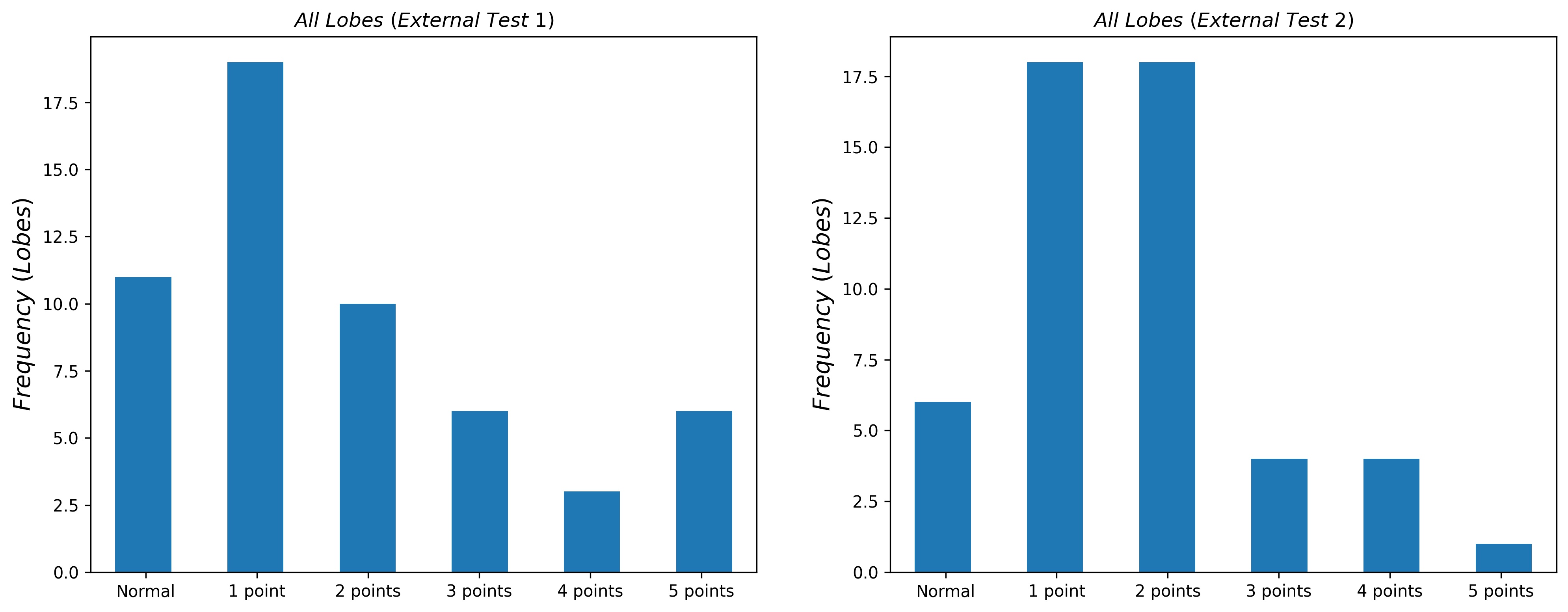

Supplement: Supplementary Figure 7 — Sample count of different classes of infection severity in External Test sets 1 and 2. [file Image_7.jpg]
